# A new era of bioclimatic extremes in the terrestrial Arctic

**DOI:** 10.1126/sciadv.adw5698

**Published:** 2026-01-07

**Authors:** Juha Aalto, Matti Kämäräinen, Mika Rantanen, Pekka Niittynen, Gareth K. Phoenix, Jonathan Lenoir, Ilya Maclean, Miska Luoto

**Affiliations:** ^1^ Climate Impacts and Adaptation, Finnish Meteorological Institute, Helsinki, Finland.; ^2^ Department of Geosciences and Geography, University of Helsinki, Helsinki, Finland.; ^3^ Department of Biological and Environmental Science, University of Jyväskylä, Jyväskylä, Finland.; ^4^ Plants Photosynthesis and Soil, School of Biosciences, University of Sheffield, Sheffield, UK.; ^5^ UMR CNRS 7058, Ecologie et Dynamique des Systèmes Anthropisés (EDYSAN), Université de Picardie Jules Verne, Amiens, France.; ^6^ Environment & Sustainability Institute, University of Exeter, Penryn Campus, Penryn, UK.

## Abstract

The Arctic climate is rapidly warming, but long-term changes in extreme weather events that cause major ecosystem disturbances are not well understood. Here, by using a state-of-the-art atmospheric reanalysis spanning the past seven decades, we show that, in many parts of the terrestrial Arctic, the frequency of extreme weather events has increased sharply. We found pronounced spatial variability in bioclimatic extremes during the past 30 years, including more droughts in the high-Arctic and greater area affected by winter-warming and rain-on-snow events, especially in the European Arctic region. Across one-third of the Arctic domain, such extreme events have only recently begun to occur. Thus, the Arctic is entering a novel era of bioclimatic extremes with likely severe consequences on cold ecosystems.

## Teaser

Long-term climate data suggest that the Arctic is entering a new era of bioclimatic extremes threatening cold ecosystems.

## INTRODUCTION

Rapid climate warming, which is amplified across the Arctic (*1*), profoundly affects the Arctic environment (*2*, *3*). Increasing mean temperatures and changing precipitation regimes have altered the cryosphere (i.e., snow, ice, and permafrost) and biosphere (e.g., carbon cycle and vegetation), affecting whole ecosystems and societies (*4*–*6*). Examples of ecosystem alterations include the overall expansion in deciduous and large stature shrubs and increased vegetation productivity, leading to the broad “Arctic greening” trend (*7*, *8*). However, an increasingly complex picture is emerging with some areas undergoing the opposite process of decline in primary productivity, termed “Arctic browning” (*8*, *9*). In tandem, Arctic species ranges are redistributing whereas Arctic community compositions are being reshuffled through a process coined “borealization” (*10*). The climatic drivers of such biome-wide megatrends can be complex but have often been linked to gradual shifts in mean climate manifesting as, for example, increasingly warm and long Arctic summers (*3*, *7*). Recently, the role of acute extreme weather events, such as rain-on-snow events (ROS) or winter-warming events (WWE) (*11*, *12*), have gained interest due to potentially severe impacts on Arctic terrestrial ecosystems, particularly on Arctic browning (*9*, *13*–*17*).

The bioclimate describes climatic conditions particularly relevant for living organisms and ecosystem functioning due to its inherent linkages to biological performance, reproduction, and survival (*18*–*20*). Thus, bioclimatic indicators, such as growing degree days at different biologically meaningful temperature thresholds or extremes, can be highly useful in, for instance, investigating species’ distributions and range dynamics across geographic scales (*21*–*23*). Existing regional and global bioclimatic datasets (*24*–*27*) chiefly summarize the bioclimate in terms of annual or seasonal temperature and precipitation means and extremes and are often aggregated over 30 years to represent long-term climatologies. However, they typically lack variables that capture the frequency or magnitude of short-term extreme weather events, such as heat and drought periods, WWE, or extreme wind speeds. This is a notable shortfall as extreme events may have drastic detrimental effects on the growth, reproduction, and survival of organisms, often pushing them beyond lethal thresholds (*28*), resulting in large-scale population crashes and vegetation diebacks (*29*, *30*). Deriving bioclimatic variables that summarize both the variability and trends in extreme events require daily or subdaily climate datasets that extend over sufficiently long time periods (*11*, *31*).

The Arctic comprises a wide spectrum of climate conditions, largely driven by extensive latitude and continentality gradients, along with pronounced intra- and interseasonal variability (*32*). As an example, northern Europe, Iceland, and southern Greenland are characterized by a maritime climate with mild winters and relatively high precipitation levels. In contrast, continental Siberia has low cumulative rainfall with a pronounced temperature seasonality. Arctic climate change has been particularly acute since the 1970s, with wintertime warming rates clearly exceeding summertime ones (*1*, *33*, *34*). Thus, changes in mean temperatures and precipitation hide important changes in the variability of many other bioclimatic variables that are based on thresholds, extremes, and other joint constraints (e.g., the concomitant occurrence of snow cover and water phase) (*3*, *35*, *36*). For example, the increase in the severity and frequency of heatwave events may be buffered in coastal areas, and ROS are more likely to occur during mild winters (e.g., in Scandinavia). As an example of biological impacts, extreme winter warming can damage not only plants’ shoots that are exposed above the insulating snow layer (*12*) but also large mammals roaming on icy grounds if ROS happen shortly before a cold spell (*37*). Until now, a complete picture of the long-term trend and variability in meaningful bioclimatic thresholds and extreme events for terrestrial life across the entire Arctic domain has been lacking. This has limited our understanding of how Arctic terrestrial organisms and ecosystems have and will respond to increasing frequency of extreme weather events, pushing Arctic biodiversity toward previously unknown and uncharted bioclimatic territories.

Here, we investigate the variability and change in 11 bioclimatic variables integrating seasonal effects and extreme weather events relevant for terrestrial life, over seven decades across the Arctic (Table 1; Materials and Methods). We build our analyses on the state-of-the-art gridded dataset of the bioclimatic atlas of the terrestrial Arctic (ARCLIM) (*31*). The ARCLIM dataset is based on the new atmospheric reanalysis of ERA5-Land of which hourly temporal resolution allows the production of a wide spectrum of bioclimatic variables that are based on seasonal thresholds [snow season length (SSL), thermal growing degree day sum (GDD), freezing degree days (FDD), and summer warmth index (SWI)], extreme weather events [frost during the growing season (FGS), number of ROS, number of WWE (i.e., increase in daily mean above +2°C during snow cover), heatwave magnitude index (HWMI), vapor pressure deficit magnitude index (VPDI), and number of high wind speed events (HWE)], and other joint constraints tailored to Arctic terrestrial ecosystems (*9*, *12*). We provide a comprehensive picture of the Arctic (>60°N, 1950 to 2022) bioclimatic variability and trends, especially highlighting the recent changes over the past 30 years. This understanding of a new era of bioclimatic dynamics will enhance our ability to predict the impacts of climate change on Arctic terrestrial ecosystems, ultimately helping human societies to develop tailored conservation actions for climate change adaptation and mitigation.

**Table 1.** Summary of the bioclimatic variables considered. Full definitions are provided in Materials and Methods.

Full name	Abbreviation	Unit
Thermal growing degree day sum	GDD	°C days
Thermal growing season length	GSL	days
Frost during the growing season	FGS	°C days
Freezing degree days	FDD	°C days
Snow season length	SSL	days
Summer warmth index	SWI	
Number of rain-on-snow events	ROS	year^−1^
Number of winter-warming events	WWE	year^−1^
Heatwave magnitude index	HWMI	
Vapor pressure deficit magnitude index	VPDI	
Number of high wind speed events	HWE	year^−1^

## RESULTS AND DISCUSSION

### Long-term trends in the Arctic bioclimate

**Fig. 1.**
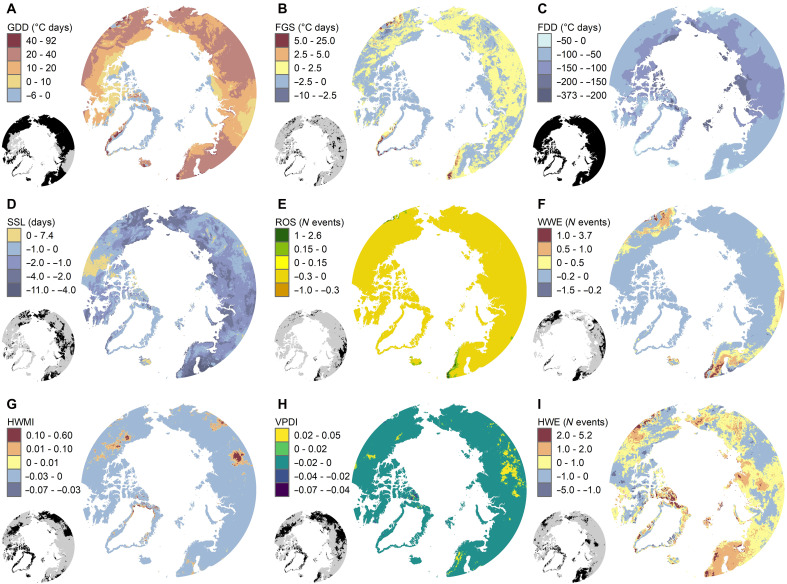
Spatial variation in long-term bioclimatic trends across the Arctic. Maps depict pixel-wise trends (1950 to 2022; the magnitude of change is expressed as per decade) at spatial resolution of 0.1° × 0.1° across a selection of bioclimatic variables. (**A**) GDD (°C days). (**B**) FGS (°C days). (**C**) FDD (°C days). (**D**) SSL (days). (**E**) ROS: number of ROS. (**F**) WWE: number of WWE. (**G**) HWMI. (**H**) VPDI. (**I**) HWE: number of HWE. Temporal trends were estimated using the nonparametric Sen’s slope method. The black areas in the small maps indicate pixels associated with statistically significant trends (*P* ≤ 0.05) tested using the Mann-Kendall trend test.

We found a pronounced spatial variability in the long-term (1950 to 2022) trends of bioclimatic variables that are tailored to capture meaningful seasonal periods and extreme events for Arctic terrestrial ecosystems (Fig. 1). As a general pattern, many of the so-called seasonally integrated variables relying on relevant threshold values for terrestrial ecosystems, such as growing and freezing degree days, showed geographically widespread changes over the past 70 years.

In contrast, trends in bioclimatic variables related to extreme weather events, such as WWE and heatwave events, tend to show less geographically widespread patterns that are instead often clustered on specific sectors of the Arctic domain, potentially responsible for some of the marked spatial variability we found in the mean bioclimate over 1991 to 2020 (fig. S1). For example, the northern European sector and continental Siberia show very consistent trends in many of the bioclimatic variables that are related to extreme events (Fig. 1), such as FGS and ROS. These pronounced geographical discrepancies contrast with more uniform geographical patterns in the long-term trends of mean annual air temperature and precipitation sum (fig. S2). This is an important finding as so far much of the research has focused either on single climatic variables (*11*), on restricted geographical extents (*12*), or on quantifying changes in bioclimatic variables characterizing annual and/or coarse seasonal averages (*22*, *38*), thus totally missing extreme weather events that may be detectable only from hourly time series.

### Recent increase in extreme weather events across the Arctic

**Fig. 2.**
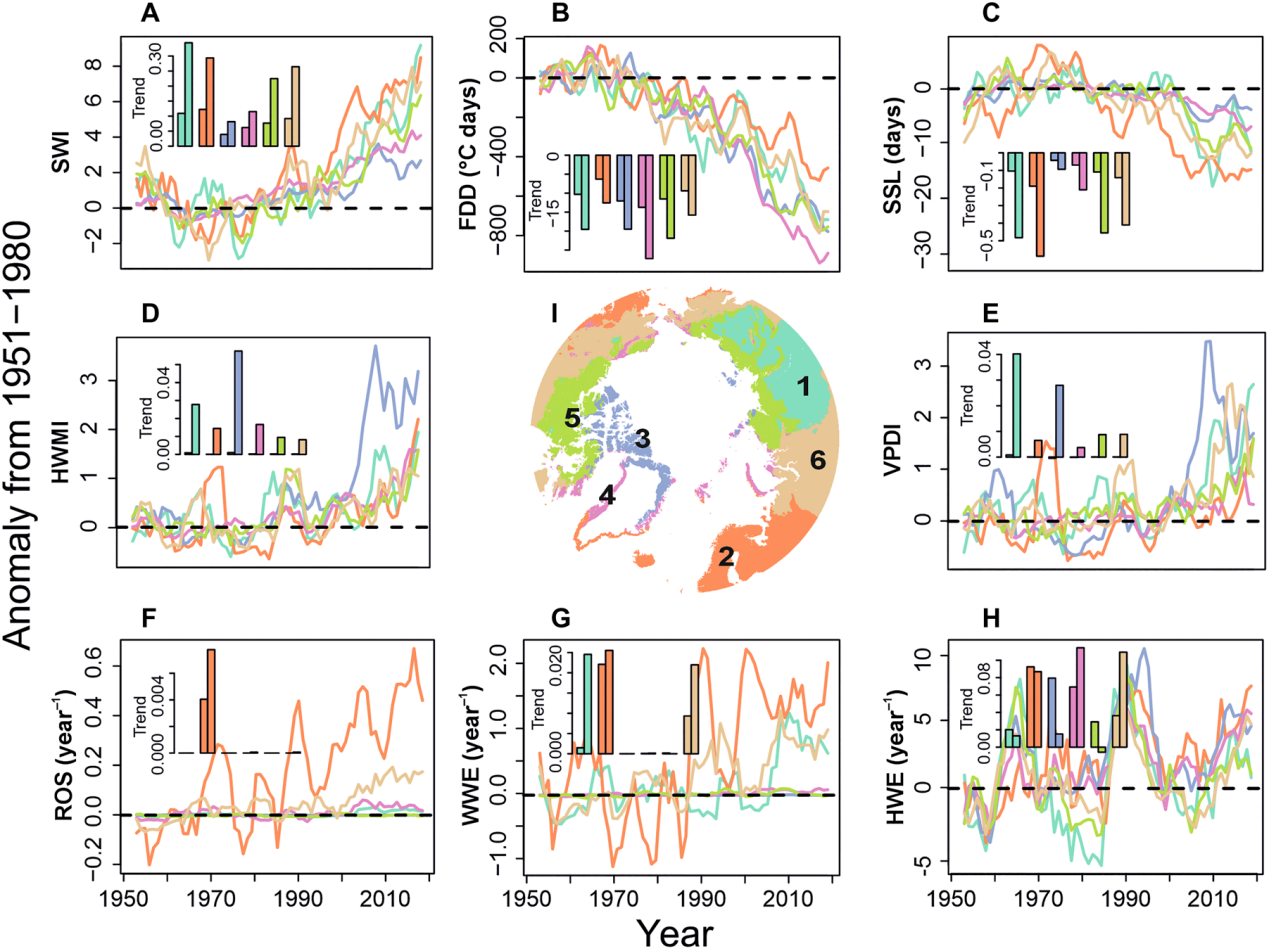
Long-term bioclimatic dynamics across the Arctic. The time series in each panel depict changes, from 1950 to 2022, for a selected set of seasonally integrated as well as for a subset of variables related to extreme weather events, computed as anomalies to the long-term historic mean of 1951 to 1980. (**A**) SWI. (**B**) FDD (°C days). (**C**) SSL (days). (**D**) HWMI. (**E**) VPDI. (**F**) Number of ROS. (**G**) Number of WWE. (**H**) Number of HWE. The annual time series have been smoothed using 5-year moving averages. The lines represent annual averages over each climate cluster [map in (**I**); see Materials and Methods‘ indicated with matching coloring. The bar plots in each panel show cluster-averaged and pixel-wise trends per year over two periods: 1950 to 2022 (first bar) and 1993 to 2022 (second bar), estimated using the nonparametric Sen’s slope method. Numerical results are presented in table S2.

Our expectation is that bioclimatic variability and long-term changes are influenced by regional climates. To unravel this, we conducted a spatial cluster analysis to first identify distinct and discrete climatic groups based on long-term (1991 to 2020) average conditions for three climatic variables: mean annual temperature, annual precipitation sum, and temperature annual range (Fig. 2 and table S1; Materials and Methods). For each of the six resulting climatic clusters and for each of the 11 studied bioclimatic variables, we then computed a time series of yearly anomalies relative to the mean of 1951 to 1980. The resulting six clusters were as follows: “Boreal continental” (cluster 1), “Warm humid coastal” (2), “High-Arctic Archipelago” (3), “Moderate coastal” (4), “Tundra coastal” (5), and “Mild Arctic” (6).

Irrespective of the climatic cluster considered and for most of the seasonally integrated and event-related bioclimatic variables studied, we found that the cluster-averaged temporal trends (Sen’s slope) over the recent 30 years (1993 to 2022) are systematically more pronounced compared to the trends calculated over the 73-year period of 1950 to 2022 (Fig. 2 and table S2). This recent and rapid increase in the magnitude of computed anomalies is particularly acute for the variables depicting heatwaves (HMWI, particularly in cluster 3), droughts (VPDI, particularly in cluster 1), and SSL, showing either more positive (i.e., higher HMWI and VPDI) or more negative (i.e., shorter SSL) anomalies. The pattern is robust and consistent across all bioclimatic variables investigated. Depending on the bioclimatic variable considered, we also found clear differences between the six climatic clusters in the magnitude of the anomalies during the recent period (1993 to 2022) (Fig. 2). For example, among the seasonally integrated bioclimatic variables, the recent decrease in FDD (Fig. 2B) has been particularly pronounced in cluster 4 [i.e., “moderate coastal” climate; mean trend: −27.3°C days/year; 90% range of variation: (−56.8 to −8.0)], on average, compared to cluster 2 (i.e., “warm humid coastal” climate) for which the magnitude of the recent mean FDD trend is more than 50% less [−12.6°C days/year (−17.3 to −6.9)]. In contrast, recent mean trends in SSL are considerably weaker in cluster 3 [“high-Arctic archipelago” climate; −0.1 days/year (−0.7 to 0.2)] and cluster 4 [“moderate coastal” climate; −0.2 days/year (−1.0 to 0.2)] compared to the other climatic clusters covering the more continental parts of the Arctic, such as cluster 1 [“Boreal continental”; mean trend: −0.5 days/year (−1.1 to 0.1)] (Fig. 2C). In general, changes in winter temperatures have been less pronounced in the northern Europe Atlantic sector compared to high-Arctic areas, which have been greatly affected by the loss of sea ice (*39*).

Overall, extreme weather events have become more frequent across the Arctic (Fig. 1, E to I). Nevertheless, our long-term time series also suggest region-specific bioclimatic changes for some of the studied extreme weather events. For example, in the northern European and Icelandic part of the Arctic domain (cluster 2), ROS events have increased throughout the study period [0.004 events/year (0.000 to 0.026)] with a slight acceleration in the mean trend during the recent decades [0.008 events/year (0.000 to 0.077) during 1993 to 2022] (Fig. 2F). However, our data suggest that ROS events are rare in other parts of the Arctic, in agreement with a previous study (*11*).

Of all the climatic clusters we considered, the Canadian Archipelago and northern Greenland have seen the fastest increase in the occurrence of strong heatwaves, but conversely, the total summer heat, as represented by the SWI, has seen the slowest increase. This region is still largely surrounded by sea ice during summer, which may prevent the temperature increase from being as rapid as in other climatic clusters, especially during early summer. However, autumn and winter warming have been particularly pronounced across this region, with existing reports of ecosystem-level impacts involving extreme weather events (*40*). Moreover, as the buffering effect of the sea ice is gradually reduced, the increase in summer temperature is likely to be reflected in high-summer heatwaves (*41*). Across the Arctic region and extending to the Circum-Arctic coastal tundra, there has been a strong and recent increase in extreme drought events. The simultaneous increase in heat and drought can exacerbate various ecosystem disturbances and harm biological functions (*8*, *42*, *43*).

### Manifold expansion of the area covered by extreme weather events

**Fig. 3.**
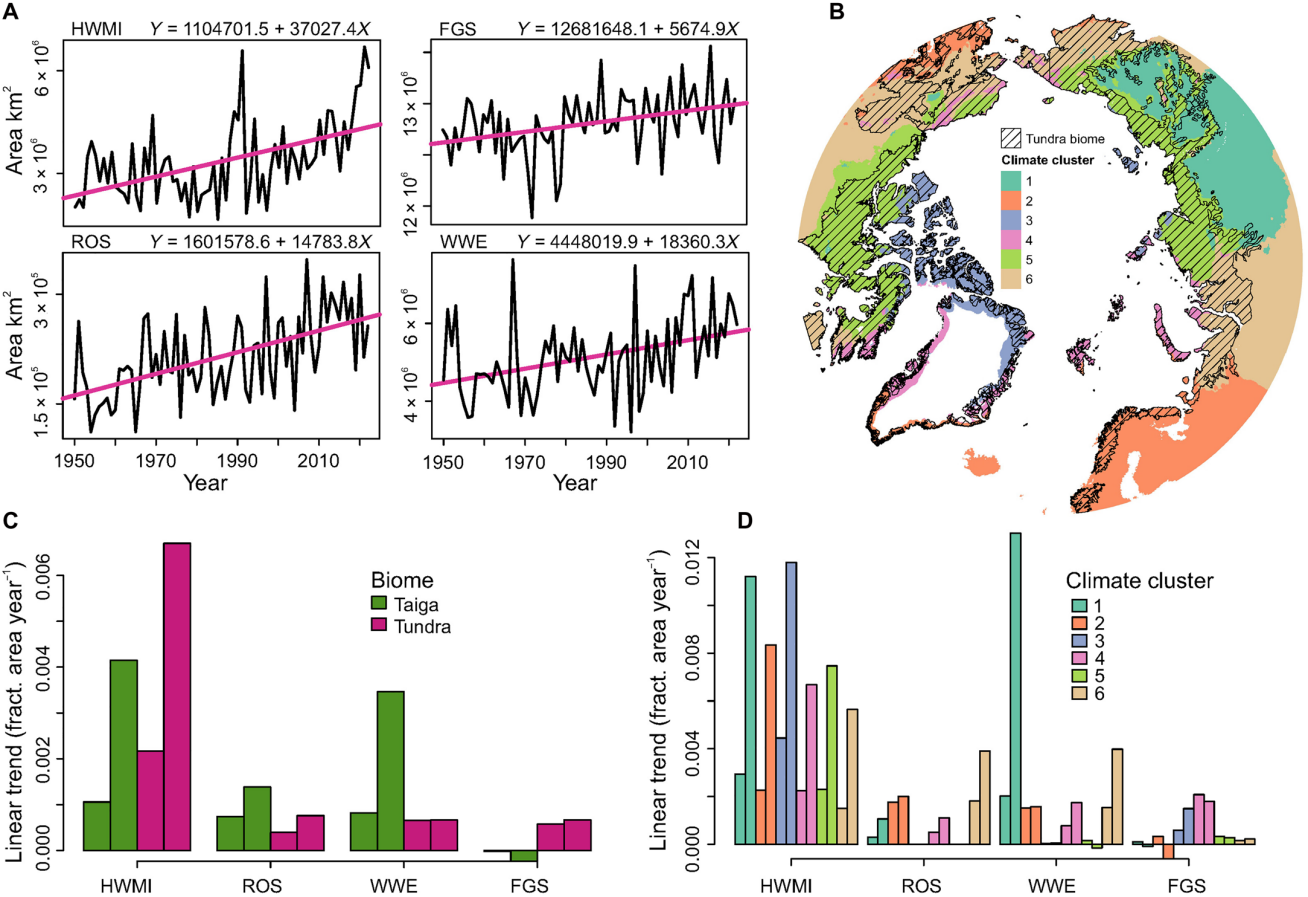
Increasing spatial coverage in extreme weather events. Temporal trends (1950 to 2022) in the total surface area covered by the occurrences of extreme weather events across the Arctic. (**A**) Annual time series of the total areas covered by HWMI, FGS, ROS, and WWE, respectively, over the entire study domain. The purple lines depict the least-squares fit, with equations provided at the top of each panel. All temporal trends are statistically significant (*P* ≤ 0.05). For HWMI, an index value threshold of ≥3 was used to compute the area, and for FGS, ROS events, and WWE, a value threshold of >0 was used. (**B**) Geographical extent of the areas where tundra is found, depicted with hatching, with the remaining area belonging to the taiga biome. Climate clusters are presented and colored as in Fig. 2. (**C**) Temporal trends in the fractional area covered by the extreme weather events in tundra and taiga biomes. The first bars indicate the estimated least-squares trend for the entire period (1950 to 2022), and the latter bars show the trend for the most recent 30-year period (1993 to 2022). Numerical results for all variables are presented in table S3. (**D**) As in (C), but the trends are grouped by the six climate clusters with the coloring of the bars matching the map in (B). Numerical results are presented in table S4.

On a yearly basis, the total area covered by heatwave (HWMI ≥ 3), drought (VPDI ≥ 3), rain-on-snow (ROS > 0), and winter-warming (WWE > 0) events has increased (*P* ≤ 0.05) by 3.4-, 3.0-, 1.7-, and 1.3-fold, respectively, during the 73-year study period (Fig. 3A and figs. S3 and S4). Similarly, the total area covered by frost events during the growing season (FGS > 0) increased over time but at a relatively smaller pace (~+3% over the 73-year period). After examining the trends for each of the two major Arctic biomes, tundra and taiga (Fig. 3, B and C, and table S3), we found that, in the Taiga biome, the recent trends in the fractional area where the events occur have been consistently higher compared to the full period. A similar pattern, but with faster changes over time, holds for the tundra biome (excluding WWE with no evident increase). Overall, these results suggest a recent increase in the exposure of Arctic terrestrial ecosystems to extreme weather events, especially so for the boreal continental climate cluster showing widespread expansion of its exposure to heatwaves, droughts, and WWE (Fig. 3D and table S4).

In addition to the tundra/taiga comparisons, bioclimatic extremes have changed differently across the Arctic depending on the regional climatology. For example, increases in the number of ROS is particularly evident across terrestrial areas under oceanic influences, which are affected by warm ocean currents with moist advection and relatively mild winters, such as Scandinavia, Iceland, and southern Alaska. However, because of recent warming and its impacts on, e.g., precipitation in either solid (i.e., hail and snowfall) or liquid (rainfall) state, areas susceptible to ROS and WWE have notably increased in geographical extent, especially in more continental regions of the Arctic.

**Fig. 4.**
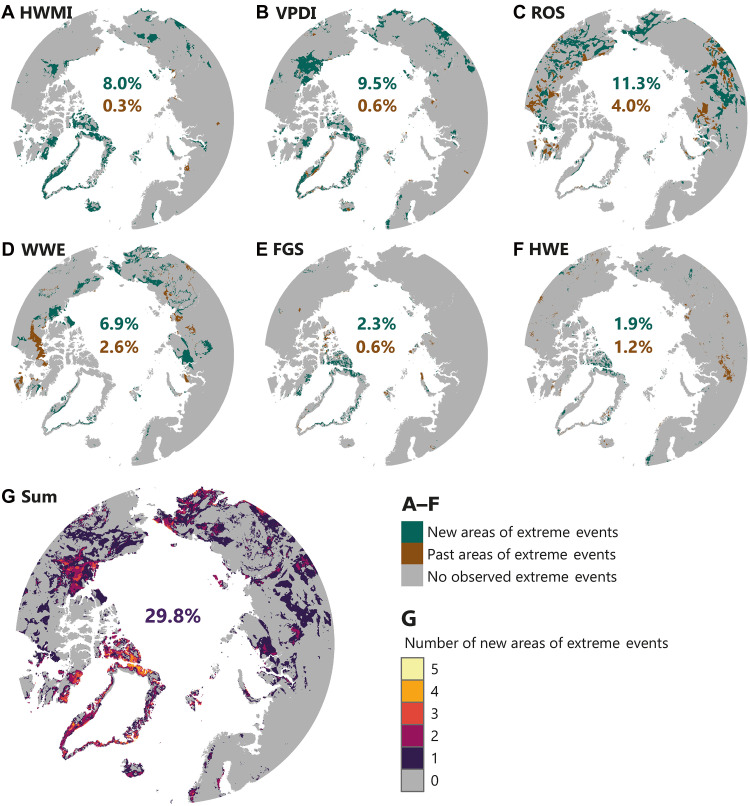
Novel and disappearing areas of extreme weather events. (**A**) HWMI (HWMI ≥ 3). (**B**) VPDI (VPDI ≥ 3). (**C**) ROS (ROS > 0). (**D**) WWE (WWE > 0). (**E**) FGS (FGS > 0). (**F**) HWE (HWE ≥ 90th percentile). Spatial units in dark green depict areas where no extreme weather events have occurred during the first 30-year period of the studied time series (1950 to 1979) but have occurred at least once during the past 30-year period of the studied time series (1993 to 2022) (i.e., the novel regimes of climatic extremes). Similarly, spatial units (0.1° × 0.1°) in brown depict areas where no extreme events have occurred during 1993 to 2022 but have occurred at least once during 1950 to 1979 (i.e., the disappearing regimes of climatic extremes). (**G**) Number of overlapping new areas of extreme events (dark green spatial units), i.e., the pixel-wise sum over (A) to (F). The percentages in (A) to (F) indicate the proportional areal coverage of the new and past areas of extreme events (green and brown colors, respectively). In (G), the percentage indicates the proportional areal coverage of any of (A) to (F) (i.e., sum equals one).

It is also apparent, and of major importance, that Arctic terrestrial ecosystems are being increasingly exposed to previously unseen extreme weather events. By identifying spatial units (0.1° × 0.1°) switching from no single extreme weather event during the first 30-year period of the studied time series (1950 to 1979, here the baseline) to at least one extreme weather event occurring during the past 30-year period of the studied time series (1993 to 2022) (i.e., “new areas of extreme events”), and vice versa (i.e., “past areas of extreme events”), we found that 29.8% of Arctic terrestrial areas have begun to be exposed to extreme weather events compared to the baseline period (Fig. 4). The largest increase in fractional area covered is for ROS (11.3% of new areas; Fig. 4C). Noteworthy, although the continental Greenland Ice Sheet was masked out from the analyses due to the focus on bioclimatic conditions relevant for terrestrial ecosystems and organisms, ROS on continental ice sheets and glaciers have been shown to have recently become more frequent and affecting their movement and evolution (*44*).

### The two dimensions of bioclimate change

**Fig. 5.**
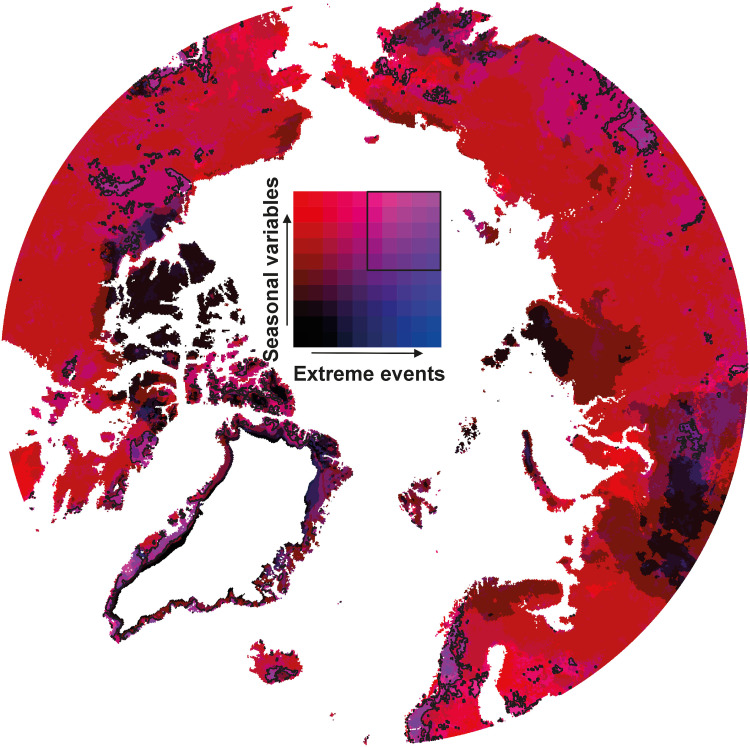
Synthesis of the temporal changes in seasonally integrated and event-related bioclimatic variables. The map depicts the pixel-wise proportion (0 to 1) of bioclimatic variables that are associated with a statistically significant change (*P* ≤ 0.05, two-sided *t* test) between the historic and modern 30-year period: 1950 to 1979 (i.e., the first 30 years of the investigated time series) versus 1993 to 2023 (i.e., the past 30 years of the investigated time series). The color scale is constructed along two orthogonal dimensions: one dimension corresponding to the set of seasonally integrated bioclimatic variables (i.e., GDD, FDD, SSL, and the SWI) and the second dimension corresponding to the set of bioclimatic variables related to extreme weather events (i.e., FGS, number of ROS, number of WWE, HWMI, VPDI, and number of HWE). The contouring delineates areas where the proportion of statistically significant changes are equal or larger than 0.5 in both seasonal and event-related variables.

Last, we analyzed temporal changes in bioclimatic conditions between the historic (1950 to 1979) and modern (1993 to 2022) period by integrating all studied bioclimatic variables into the bidimensional space of seasonal variables against variables related to extreme weather events. We found that temporal changes in seasonal variables are widespread and dominate across most of the Arctic domain (Fig. 5). In contrast, temporal increases in extreme weather events are more localized and clustered, for example, in western Scandinavia, coastal Greenland, the Canadian high-Arctic Archipelago, and Central Siberia. Hence, considering that these areas also experience important temporal changes in seasonal variables, they can be highlighted as “hotspots” of observed bioclimate changes potentially meaningful for explaining local trends in biodiversity changes (*45*, *46*).

The results indicate that an increasingly large part of terrestrial ecosystems across the Arctic are facing higher exposure to extreme weather events and, in many areas, some of the extreme events have only recently begun to emerge. This previously unknown regime of extreme weather events suggests that many Arctic ecosystems are now facing unprecedented weather conditions to which the biota may not be adapted. Moreover, our results highlight a few distinct areas, namely, Scandinavian mountains, coastlines of Greenland, and the Canadian high Arctic archipelago, where a multitude of both seasonal variables and variables related to extreme weather events showed significant increasing trends during the study period.

Recent acceleration in temporal changes of many of the studied variables stems from the general increase in the pace of climate warming since the 1970s (*1*). Although the observed changes in bioclimate indicators are consistent with ongoing anthropogenic climate change, we did not conduct a formal attribution analysis to identify the specific causes. Thus, some of the changes may also be due to internal climate variability. For example, it is known that internal climate variability (i.e., the so-called positive Arctic mode) enhanced an Arctic amplification trend during 1980 to 2022 (*47*), which may have positively contributed to some of the 30-year trends we reported in our study. Furthermore, large-scale atmospheric circulation can influence the occurrence of bioclimatic extremes across the Arctic. For example, it has been observed that a positive phase of the Arctic Oscillation (AO) favors the occurrence of wildfires in Siberia (*48*, *49*), most likely triggered by extreme weather events acting as catalyzers for wildfires and that can be tracked by focusing on variables such as HWMI and VPDI. Large-scale circulation can also modulate long-term trends. For example, the cyclical behavior of HWE observed in this study (i.e., peaking in the early 1990s and declining thereafter) may be partly due to the peak in the AO during the early 1990s (*50*). A positive AO favors higher cyclone activity in the European sector of the Arctic, for example, in Svalbard (*51*), thus increasing the risk of high wind speeds, at least in this region. These changes, in turn, can affect terrestrial ecosystems as wind can not only affect plant dispersal through seed and spore transportation but also trigger local desiccation and windchill to Arctic biota (*52*, *53*).

This study is based on bioclimatic variables from the ARCLIM dataset (*31*), which has been derived from the ERA5-Land dataset (*54*). Note that ERA5-Land is a downscaled reanalysis of the land component of the global ERA5 dataset (*55*). In ERA5, all available in situ surface weather observations have been assimilated along with a wide range of remote sensing data. This improves the accuracy of the ARCLIM variables in high-latitude regions where the network of in situ weather stations is relatively sparse. Nevertheless, Rantanen *et al.* (*31*) found still modest biases in summer and winter mean temperatures in ERA5-Land when compared to field measurements from weather stations. These biases may affect those bioclimatic variables, such as growing degree days or WWE, that are based on absolute threshold values. Because the reliability of ERA5-Land depends strongly on the availability of field observations, it is also important to note that uncertainties are expected to be higher before the satellite era.

Here, we have shown that the Arctic has entered a new era of bioclimatic extremes. These results provide a benchmark to improve our current understanding of how climate change affects climatic conditions that govern ecosystem and ecological processes and biological functions in cold-climate regions. Our results are valuable for interpreting observed trends in biodiversity changes and predicting future ecosystem changes across the Arctic.

## MATERIALS AND METHODS

### General data processing

The study uses a variety of geospatial data sources, which were managed using geographical information systems, namely, ArcGIS Spatial Analyst tools, and R statistical programming environment (*56*) with the raster functionalities of the terra library (*57*). Area calculations were conducted using terra functions cellSize() and expanse() that account for the changing surface areas of the pixels by latitude.

### ARCLIM bioclimatic indicators

The bioclimatic variables were derived from the ERA5-Land reanalysis (*54*) and downloaded from the ARCLIM dataset (*31*). The original ARCLIM covers the years 1950 to 2021, but here the dataset was extended by 1 year to cover 1950 to 2022. The ERA5-Land reanalysis is produced by the European Centre for Medium-Range Weather Forecasts. ERA5-Land is essentially a downscaled land component of the ERA5 reanalysis (*55*) and forced by the ERA5 meteorological fields. Thus, observations are not directly assimilated to ERA5-Land, but the observations influence the land surface evolution via the atmospheric forcing (*54*). ERA5-Land has a horizontal resolution of 0.1° and provides hourly data on meteorological variables at the land surface.

The ARCLIM dataset consists of 14 seasonally integrated and event-type variables that are particularly relevant for studying the changes in the Arctic ecosystems. In this study, the bioclimatic variables were analyzed over a domain ≥ 60°N and at the spatial resolution of 0.1° × 0.1°. For visualizations, the data were projected into the Polar Stereographic coordinate reference system (epsg: 3995). Short definitions of the bioclimatic variables are given below, but further information can be found from Rantanen *et al.* (*31*).

1) Thermal growing season length (GSL): the period of the year when the daily mean temperature stays at or over a 5°C threshold (*58*).

2) GDD: the sum of daily mean temperatures, which exceed the 5°C threshold during the growing season (*58*).

3) FGS: the sum of daily minimum skin temperatures, which are below freezing during the growing season.

4) FDD: the sum of daily mean temperatures, which are below freezing during the winter season.

5) ROS: days with the total liquid precipitation greater than 5 mm on a snow-covered grid cell (*11*, *59*).

6) WWE: days when the daily mean temperature of 2°C or higher occurs on a snow-covered grid cell (*12*).

7) HWMI: cumulative index, which takes into account both the intensity and duration of the strongest heatwave of the summer (*60*).

8) VPDI: VPDI is similar to HWMI but calculated for vapor pressure deficit.

9) SWI: annual sum of monthly mean 2-m temperatures above 0°C (*7*).

10) SSL: the longest continuous period of the year when the grid cell is snow covered.

11) HWE: the annual number of days when the 10-m height daily maximum wind speed in the grid cell exceeds the 90th percentile threshold.

The entire analysis presented here is based on the ERA5-Land reanalysis, which is produced by combining all available in situ and remote sensing observations with a global numerical weather prediction model. In situ observations from weather stations are therefore not in perfect agreement with the ERA5-Land data, although they are generally very close to each other. For example, the correlation between summer mean temperature from in situ observations and ERA5-Land data at Arctic terrestrial weather stations was 0.94 (*31*).

The indicators presented in this paper are defined in the ARCLIM dataset (*31*). We acknowledge that the list of indicators is not exhaustive and that the definitions of the indicators may limit their occurrence to a specific part of the Arctic. For example, WWE is defined using a fixed threshold of 2°C for the daily mean temperature during November to April in a snow-covered grid cell. Daily mean temperatures of 2°C, and thus WWE, are very rare in the High Arctic or Eastern Siberia during the winter season. Comparing different extreme weather events is therefore very difficult for the entire Arctic region as the definition of extreme varies in place and time. For this study, the Greenland Ice Sheet was masked out from the analyses due to our focus on bioclimatic conditions relevant to terrestrial ecosystems and organisms.

### Biome classification

The distribution of the two major biomes (i.e., taiga and tundra) were extracted from the Global Ecoregions dataset (*61*). The original vector dataset was converted into a raster format and resampled (using nearest neighbor interpolation) to the matching extent and resolution with the ARCLIM data.

### Climate clustering

To characterize and classify the climate over the Arctic, the KMeans clustering algorithm (*62*) was applied to three climate variables: the annual precipitation sum, the annual mean temperature, and the annual maximum monthly mean temperature difference. Before feeding these data to the classification algorithm, they were aggregated as pixel means over the period 1991 to 2021, and Gaussian normalization was applied to each variable to indirectly assign them a similar weight in clustering. The KMeans algorithm numbers the clusters randomly, and the numbering of the clusters of this study follows the random order of the algorithm. Various numbers of clusters were tested, but six was found to be the best compromise: Increasing the number of clusters would create more spatially heterogeneous clusters and more complicated analysis, and decreasing the number would hide a large amount of climate variability inside the large land masses of the few clusters.

Table S1 shows the cluster means and 98% variability ranges inside them from the raw, unnormalized variables, and fig. S5 depicts the spatial distribution of the clustering variables after normalization. Pearson’s correlation coefficients were calculated between the clustered variables to identify how closely they are related. The strongest pairwise correlation (0.77) was found between the annual temperatures and annual precipitation, which affects the clustering results by concentrating the cluster formation along these two axes and by slightly overshadowing the effect from the third variable (intra-annual temperature difference).

The largest annual temperature variability characterizes the first cluster (“Boreal Continental”), located in the inner eastern part of the Eurasian Continent. The annual temperatures and precipitation amounts are moderate compared to other regions. The second cluster (“Warm Humid Coastal”) can be found in the western coastal regions of Eurasia and North America, Iceland, and over the southernmost coast of Greenland. Warm and humid air masses advected from the upstream Atlantic and Pacific Oceans contribute to the high mean temperature and precipitation of this cluster. The Canadian Arctic Archipelago and the northernmost coast of Greenland are located in the driest and coldest third cluster (“High-Arctic Archipelago”). The moderate precipitation amounts and temperatures of the fourth cluster (“Moderate Coastal”) makes that cluster spatially the most diverse as these values can be found in various, mostly coastal locations and as the clustering did not get spatial information to enhance spatial closeness of the pixels. The fifth cluster (“Tundra Coastal”) represents quite closely the tundra biome over the continental North America and Eurasia (Fig. 3B). The sixth cluster (“Mild Arctic”) is the transition zone between the “Warm Humid Coastal” cluster and the “Tundra Coastal” over both continents.

### Temporal trend analysis

The magnitude and statistical significance of the trends in bioclimatic variables were estimated using the Sen’s slope method (*63*) and Mann-Kendall trend test (*64*), respectively. For pixel-wise analysis, we used the R function raster.kendall() as implemented in the R package spatialEco (*65*). The trends were estimated from the annual ARCLIM layers over two periods: 1950 to 2022 and 1993 to 2022. To estimate temporal trends in fractional areas covered by the two major biomes and the six climate clusters, simple linear regression was used.

### Synthesis of the bioclimatic changes

To synthesize the bioclimatic changes across the variables (seasonally integrated variables: SSL, GDD, FDD, and SWI; extreme events: FGS, number of ROS, number of WWE, HWMI, VPDI, and HWE), the statistical significance of the changes of the bioclimatic variables were analyzed between two 30-year periods representing the first and last parts of the entire study period: 1950 to 1979 and 1993 to 2022. The statistical significance of the change was tested in a pixel-wise manner using two-sided *t* tests, and the resulting *P* values were recoded as 1 (*P* ≤ 0.05) and 0 (*P* > 0.05). Then, the proportion (0 to 1) of significant changes over all seasonally integrated and extreme-event variables were calculated and presented as an RGB image.
