# Supplementary material for: A new era of bioclimatic extremes in the terrestrial Arctic
Source: Sci Adv. 2026 Jan 7;12(2):eadw5698. doi: 10.1126/sciadv.adw5698 (PMC12778043; doi:10.1126/sciadv.adw5698)
Supplement: Supplementary file 1 — Figs. S1 to S5 Tables S1 to S4 [file sciadv.adw5698_sm.pdf]

Science Advances
AAAS

Supplementary Materials for

A new era of bioclimatic extremes in the terrestrial Arctic

Juha Aalto *et al.*

Corresponding author: Juha Aalto, juha.aalto@fmi.fi

Sci. Adv. **12**, eadw5698 (2026)
DOI: 10.1126/sciadv.adw5698

This PDF file includes:

Figs. S1 to S5
Tables S1 to S4

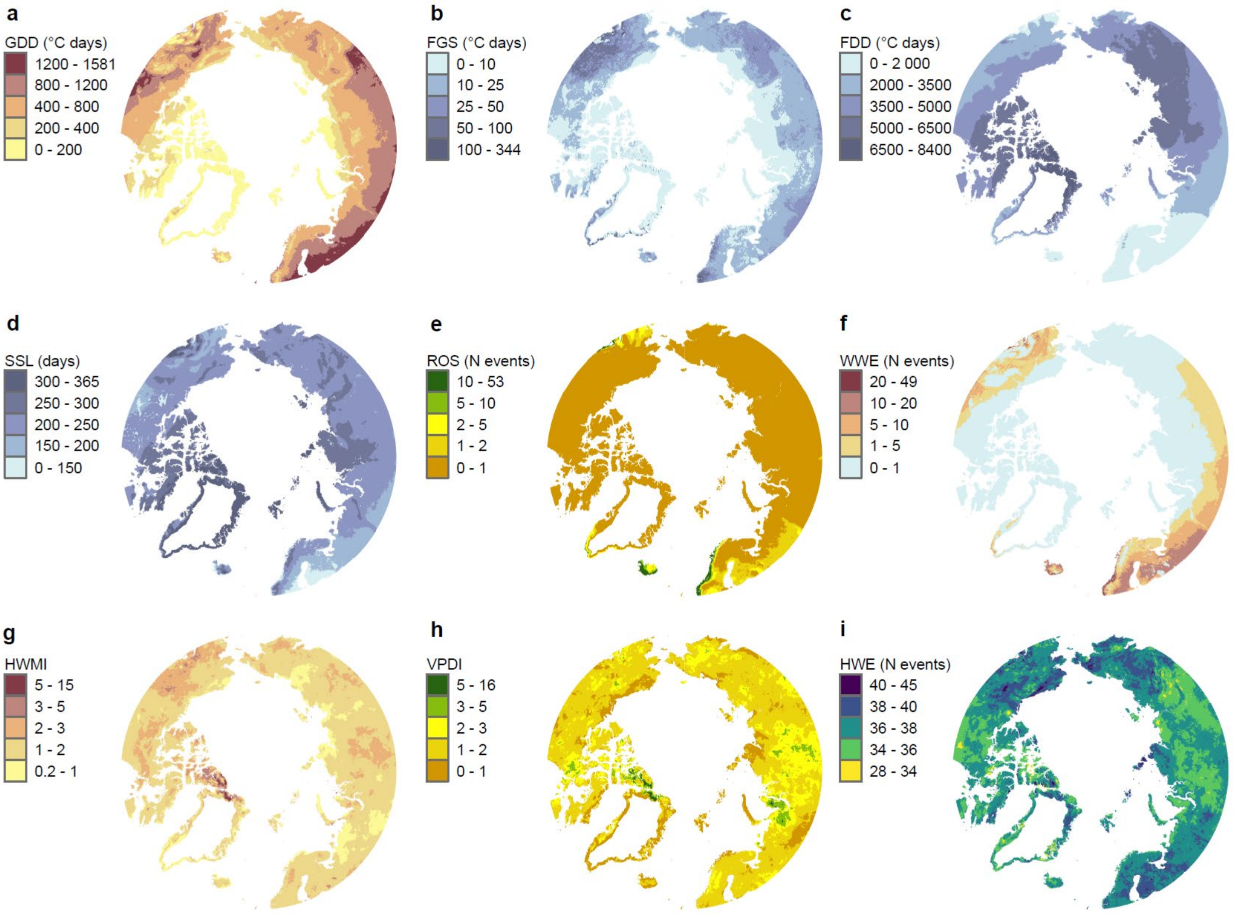

Fig. S1. The 1991–2020 averages of the selected bioclimatic variables. a *GDD*: Thermal growing degree day sum (°C days). **b** *FGS*: Frost during the growing season (°C days). **c** *FDD*: Freezing-degree days (°C days). **d** *SSL*: Snow season length (days). **e** *ROS*: Number of rain-on-snow events. **f** *WWE*: Number of winter-warming events. **g** *HWMI*: Heatwawe magnitude index. **h** *VPDI*: Vapour pressure deficit magnitude index. **i** *HWE*: Number of high wind speed events.

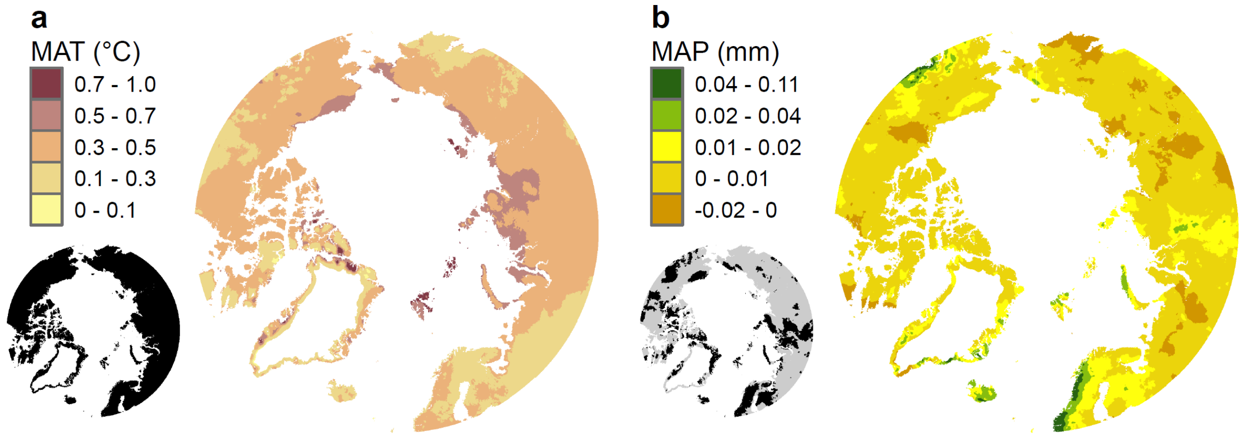

Fig. S2. Spatial variation in temporal bioclimatic trends. Maps depicting pixel-wise trends (1950–2022, expressed as per decade) in **a** mean annual air temperature (*MAT*, °C), and **b** mean annual precipitation sum (*MAP*, mm). Trends were quantified using non-parametric Sen's slope method. The black areas in the small maps indicate pixels associated with statistically significant trends ($P \leq 0.05$) assessed using the Mann-Kendall trend test.

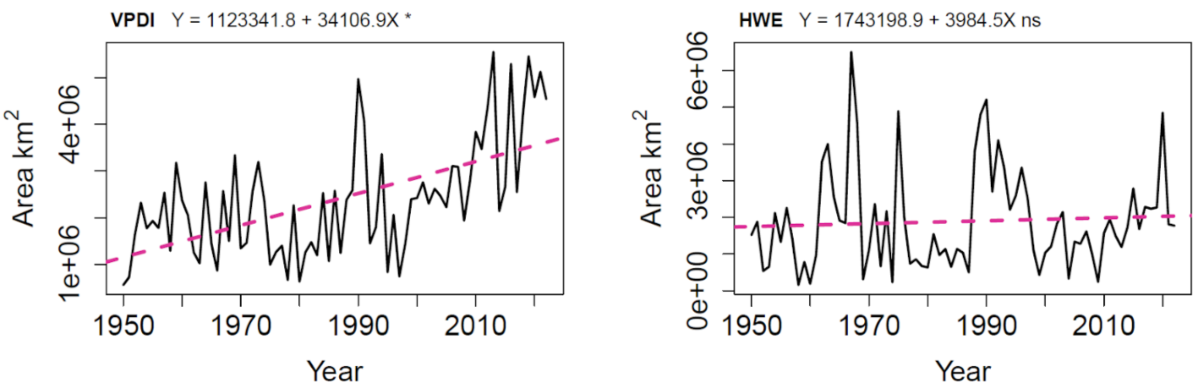

Fig. S3. Temporal trends in absolute areas covered by vapour pressure deficit magnitude index (*VPDI*) and high wind speed events (*HWE*, 90th percentile of the pixel-wise distribution), respectively, over the entire study domain. The pink dashed lines depict least-squares fit. Statistical significance of the fit is indicated with '*' ($P \leq 0.05$), whereas 'ns' depicts a non-significant trend ($P > 0.05$). For *VDPI* index value threshold of ≥ 3 was used.

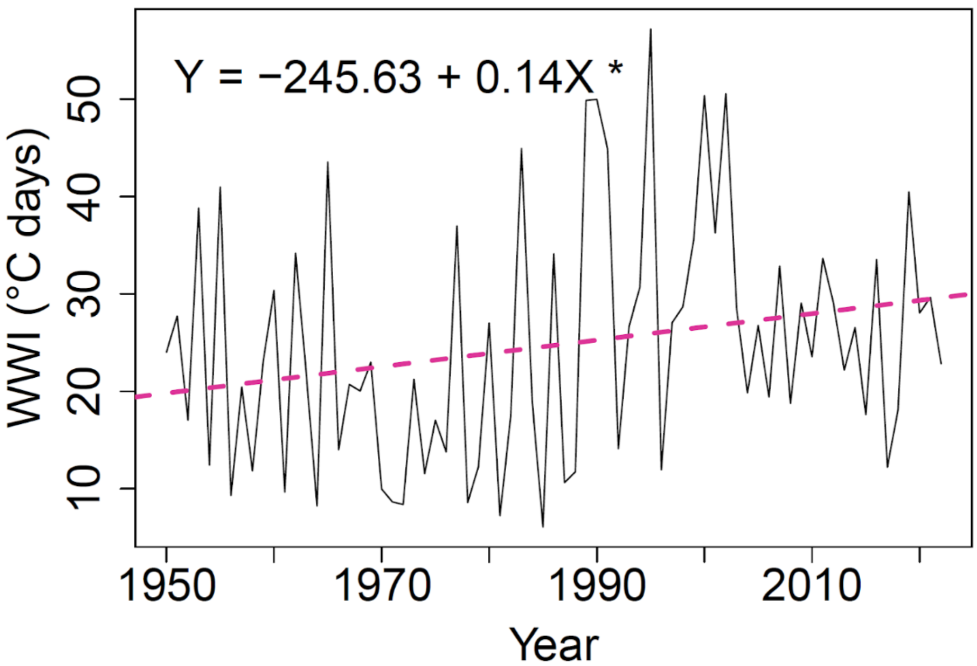

Fig. S4. Temporal trend in intensity of winter-warming events. The black line depicts annual variability in area-weighted mean intensity of winter-warming events (*WWI*, °C days) calculated over pixels where at least one winter-warming event has occurred each year. The pink dashed lines depict least-squares fit. Statistical significance of the fit is indicated with '*' ($P \leq 0.05$). To calculate the intensity of winter-warming events, the cumulative daily mean air temperature (°C) is linearly weighted by the duration throughout the winter-warming event: for example, for a 3-day event with daily mean air temperatures of 4 °C, 6 °C and 3 °C, the intensity is defined as $(4 \times 1) + (6 \times 2) + (3 \times 3) = 25$°C days.

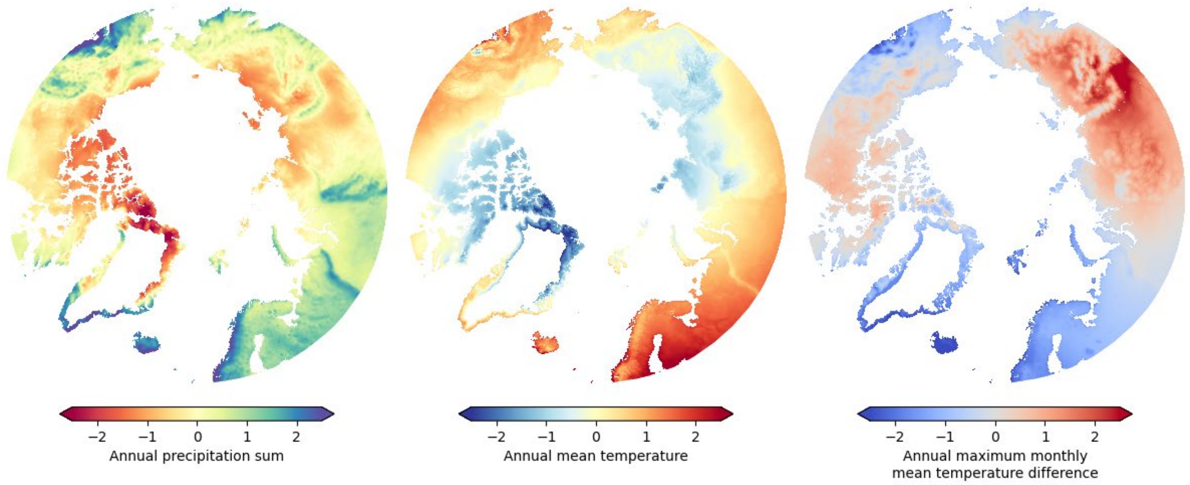

Fig. S5. Gaussian-normalized variables used for clustering. First, the daily data were aggregated to annual sums (precipitation, left) and means (temperature, center), and monthly means of temperature and the difference between the coldest and warmest month were calculated (temperature difference, right). After that the means over 1991–2020 were derived for the three variables, and finally the normalization was applied.

Table S1. Variability of clustering variables inside each cluster. The mean and the 1% and 99% extremes of the non-standardized variables over the pixels of each cluster. Values are calculated from the pixel means over the period 1991–2020.

Cluster	Annual precipitation sum [mm]			Annual mean temperature [°C]			Annual maximum monthly mean temperature difference [°C]		
	1%	Mean	99%	1%	Mean	99%	1%	Mean	99%
**1 Boreal Continental**	274	406	648	-13.5	-9.7	-4.5	46.2	51.5	61.1
**2 Warm Humid Coastal**	572	907	2799	-9.3	0.0	5.8	13.5	27.2	36.9
**3 High-Arctic Archipelago**	107	226	374	-21.3	-16.6	-12.4	26.0	35.6	44.7
**4 Moderate Coastal**	299	498	888	-17.1	-10.8	-4.9	20.2	29.4	39.2
**5 Tundra Coastal**	235	361	555	-15.3	-11.6	-5.4	36.3	43.7	49.1
**6 Mild Arctic**	354	570	895	-11.1	-5.6	-0.0	32.1	40.5	47.9

Table S2. Temporal bioclimatic trends by climate clusters. Cluster-averaged pixel-wise trends per year over two periods: 1950–2022 (i.e., "Full") and 1993–2022 (i.e., "Recent"), estimated using the Sen's slope method. For each variable cluster the mean and 5th and 95th percentiles are presented. *GSL*: growing season length (days), *GDD*: growing degree days (°C days), *FGS*: frost during growing season (°C days), *FDD*: freezing degree days (°C days), *ROS*: number of rain-on-snow events, *WWE*: number of winter-warming events, *WWI*: intensity of winter-warming events (°C days), *HWMI*: heat wave magnitude index, *VDPI*: vapour pressure deficit magnitude index, *SWI*: Summer warmth index, *SSL*: snow cover duration (days), *HWE*: number of high wind speed events, *TAVG*: mean annual air temperature (°C), *PRA*: mean annual precipitation sum (m), *SFA*: annual snowfall (m), *WSA*: annual 10-m wind speed (ms^{-1}).

Cluster	Period	Statistic	GSL	GDD	FGS	FDD	ROS	WWE	WWI	HWMI	VPDI	SWI	SSL	HWE	TAVG	PRA	SFA	WSA
1	Full	5 %	0.077	1.872	-0.106	-13.001	0.000	0.000	0.000	0.000	0.000	0.088	-0.200	-0.059	0.031	0.000	0.000	-0.001
1	Full	Mean	0.152	2.439	0.021	-10.308	0.000	0.001	0.004	0.001	0.001	0.109	-0.104	0.020	0.039	0.000	0.000	0.000
1	Full	95 %	0.240	3.038	0.115	-8.070	0.000	0.017	0.027	0.007	0.007	0.131	0.000	0.103	0.049	0.001	0.000	0.001
1	Recent	5 %	0.000	3.340	-0.586	-28.610	0.000	0.000	0.000	0.000	0.000	0.183	-1.087	-0.250	0.052	-0.002	-0.001	-0.003
1	Recent	Mean	0.493	7.566	-0.037	-19.549	0.000	0.025	0.033	0.028	0.040	0.344	-0.483	0.013	0.081	0.000	0.000	0.000
1	Recent	95 %	1.070	11.103	0.411	-10.968	0.000	0.143	0.224	0.104	0.146	0.515	0.077	0.400	0.112	0.002	0.001	0.004
2	Full	5 %	0.000	0.000	-0.081	-8.806	0.000	0.000	-0.078	0.000	0.000	0.061	-0.524	-0.042	0.023	0.000	-0.001	-0.001
2	Full	Mean	0.274	2.338	0.063	-6.302	0.004	0.022	0.098	0.000	0.000	0.122	-0.190	0.092	0.030	0.001	0.000	0.000
2	Full	95 %	0.607	3.805	0.400	-4.291	0.026	0.097	0.494	0.000	0.000	0.197	0.000	0.200	0.040	0.004	0.001	0.002
2	Recent	5 %	0.000	0.000	-0.551	-17.257	0.000	-0.056	-1.249	0.000	0.000	0.083	-1.375	-0.182	0.034	0.000	-0.003	-0.004
2	Recent	Mean	0.507	6.248	-0.024	-12.554	0.008	0.026	-0.196	0.014	0.006	0.294	-0.588	0.087	0.060	0.003	0.000	0.000
2	Recent	95 %	1.143	10.206	0.608	-6.866	0.077	0.154	0.366	0.069	0.040	0.476	0.000	0.400	0.080	0.007	0.002	0.003
3	Full	5 %	0.000	-0.187	0.000	-19.958	0.000	0.000	0.000	0.000	0.000	-0.011	-0.220	-0.067	0.015	0.000	0.000	0.000
3	Full	Mean	0.031	0.263	0.001	-12.050	0.000	0.000	0.000	0.001	0.000	0.039	-0.043	0.079	0.038	0.001	0.000	0.002
3	Full	95 %	0.220	2.021	0.000	-4.717	0.000	0.000	0.000	0.008	0.000	0.189	0.022	0.273	0.066	0.001	0.001	0.005
3	Recent	5 %	0.000	-0.484	0.000	-45.196	0.000	0.000	0.000	0.000	0.000	0.000	-0.700	-0.353	0.021	-0.001	-0.001	-0.006
3	Recent	Mean	0.086	0.594	0.006	-19.484	0.000	0.000	0.000	0.058	0.028	0.081	-0.093	0.015	0.056	0.000	0.000	0.000
3	Recent	95 %	0.600	3.995	0.000	-6.384	0.000	0.000	0.000	0.249	0.153	0.271	0.182	0.400	0.132	0.002	0.002	0.006
4	Full	5 %	0.000	0.000	-0.027	-27.709	0.000	0.000	0.000	0.000	0.000	-0.001	-0.276	-0.070	0.019	0.000	0.000	-0.002
4	Full	Mean	0.104	0.641	0.022	-13.733	0.000	0.000	0.000	0.000	0.000	0.062	-0.070	0.069	0.045	0.001	0.001	0.002
4	Full	95 %	0.516	2.415	0.146	-5.659	0.000	0.000	0.000	0.002	0.000	0.164	0.000	0.213	0.081	0.002	0.002	0.008
4	Recent	5 %	0.000	0.000	-0.182	-56.871	0.000	0.000	0.000	0.000	0.000	0.000	-1.000	-0.250	0.029	-0.002	-0.002	-0.005
4	Recent	Mean	0.183	1.353	0.013	-27.236	0.000	0.000	0.002	0.017	0.004	0.115	-0.210	0.114	0.082	0.002	0.000	0.003
4	Recent	95 %	1.178	6.711	0.220	-8.004	0.000	0.000	0.000	0.074	0.027	0.425	0.222	0.476	0.153	0.005	0.003	0.011
5	Full	5 %	0.000	0.153	-0.069	-16.395	0.000	0.000	0.000	0.000	0.000	0.025	-0.250	-0.075	0.028	0.000	0.000	-0.002
5	Full	Mean	0.179	1.363	0.004	-11.527	0.000	0.000	0.000	0.000	0.000	0.077	-0.109	0.029	0.040	0.000	0.000	0.000
5	Full	95 %	0.417	2.304	0.092	-6.957	0.000	0.000	0.000	0.000	0.000	0.121	0.061	0.136	0.053	0.001	0.000	0.003
5	Recent	5 %	0.000	-0.370	-0.248	-44.369	0.000	0.000	0.000	0.000	0.000	0.034	-1.071	-0.357	0.010	-0.001	-0.002	-0.011
5	Recent	Mean	0.616	3.787	0.006	-21.883	0.000	0.000	0.000	0.009	0.009	0.225	-0.455	-0.006	0.072	0.001	0.000	-0.003
5	Recent	95 %	1.545	9.250	0.366	-2.799	0.000	0.000	0.000	0.058	0.064	0.496	0.000	0.381	0.136	0.003	0.001	0.004
6	Full	5 %	0.055	0.825	-0.116	-13.595	0.000	0.000	0.000	0.000	0.000	0.059	-0.286	-0.047	0.026	0.000	-0.001	-0.001
6	Full	Mean	0.185	1.858	0.013	-9.376	0.000	0.009	0.035	0.000	0.000	0.092	-0.141	0.036	0.036	0.001	0.000	0.000
6	Full	95 %	0.365	3.007	0.135	-5.504	0.000	0.056	0.232	0.000	0.000	0.131	0.022	0.133	0.049	0.001	0.001	0.002
6	Recent	5 %	0.000	0.773	-0.394	-27.374	0.000	0.000	-0.143	0.000	0.000	0.063	-1.000	-0.200	0.002	-0.001	-0.001	-0.005
6	Recent	Mean	0.474	5.374	0.021	-15.763	0.000	0.022	0.064	0.008	0.009	0.264	-0.410	0.109	0.065	0.002	0.000	0.000
6	Recent	95 %	1.091	9.048	0.514	-0.139	0.000	0.190	0.584	0.041	0.051	0.455	0.143	0.500	0.111	0.005	0.002	0.004

Table S3. Increasing spatial coverage of the extreme weather events across the biomes. Temporal trends (per year) in the fractional area covered by the selected extreme weather event variables group by the two biomes; taiga and tundra. The two time periods, i.e., "Full" and "Recent", depict 1950–2022 and 1993–2022, respectively. *HWMI*: heat wave magnitude index (threshold ≥ 3), *ROS*: number of rain-on-snow events (threshold ≥ 1), *WWE*: number of winter-warming events (threshold ≥ 1), *FGS*: frost sum during growing season (threshold ≥ 1), *VDPI*: vapour pressure deficit magnitude index (threshold ≥ 3), *HWE*: number of high wind speed events (threshold ≥ 90th percentile).

Variable	$Taiga_{Full}$	$Taiga_{Recent}$	$Tundra_{Full}$	$Tundra_{Recent}$
HWMI	0.0011	0.0041	0.0022	0.0067
ROS	0.0007	0.0014	0.0004	0.0008
WWE	0.0008	0.0035	0.0007	0.0007
FGS	0.0000	-0.0002	0.0006	0.0007
VPDI	0.0012	0.0041	0.0019	0.0052
HWE	0.0001	-0.0004	0.0003	0.0000

Table S4. Increasing spatial coverage of extreme weather events across climate clusters. Temporal trends (per year) in the fractional area covered by the selected extreme weather event variables group by the climate clusters (CL). The two time periods, i.e., "Full" and "Recent", depict 1950–2022 and 1993–2022, respectively. *HWMI*: heat wave magnitude index (threshold ≥ 3), *ROS*: number of rain-on-snow events (threshold ≥ 1), *WWE*: number of winter-warming events (threshold ≥ 1), *FGS*: frost sum during growing season (threshold ≥ 1), *VDPI*: vapour pressure deficit magnitude index (threshold ≥ 3), *HWE*: number of high wind speed events (threshold ≥ 90th percentile).

Variable	$CL1_{Full}$	$CL1_{Recent}$	$CL2_{Full}$	$CL2_{Recent}$	$CL3_{Full}$	$CL3_{Recent}$	$CL4_{Full}$	$CL4_{Recent}$	$CL5_{Full}$	$CL5_{Recent}$	$CL6_{Full}$	$CL6_{Recent}$
HWMI	0.0029	0.0112	0.0023	0.0083	0.0044	0.0118	0.0023	0.0067	0.0023	0.0075	0.0015	0.0056
ROS	0.0003	0.0011	0.0018	0.0020	0.0000	0.0000	0.0005	0.0011	0.0000	0.0000	0.0018	0.0039
WWE	0.0020	0.0130	0.0015	0.0016	0.0000	0.0001	0.0008	0.0017	0.0002	-0.0001	0.0015	0.0040
FGS	0.0001	-0.0001	0.0003	-0.0006	0.0006	0.0015	0.0021	0.0018	0.0003	0.0003	0.0002	0.0002
VPDI	0.0034	0.0137	0.0017	0.0048	0.0022	0.0071	0.0010	0.0036	0.0021	0.0067	0.0020	0.0052
HWE	-0.0001	-0.0028	0.0011	0.0001	0.0009	-0.0036	0.0007	0.0021	-0.0002	-0.0006	0.0000	0.0013
